# Health Impacts of Restrictive Migration Policies: A Qualitative Study of Highly Educated Iranian Immigrants and International Students in the U.S.

**DOI:** 10.1007/s10903-025-01760-4

**Published:** 2025-08-11

**Authors:** Sarvenaz Taridashti, Mitra Naseh, Elvira M. Zamora Garcia, Jihye Lee

**Affiliations:** 1 Montclair State University, Montclair, USA; 2 Washington University in St. Louis, St. Louis, USA

**Keywords:** Iranian immigrants, Restrictive migration policies, Family separation, Emotional toll, Social determinants of health

## Abstract

Immigrants constitute a substantial portion of the United States population and represent a significant minoritized group whose health is impacted by the country’s migration policies as social determinants of health. This qualitative study used a phenomenological approach to document and explore the lived experiences of highly educated Iranian immigrants and International Students within the context of U.S. migration policies while considering the Social Determinants of Health framework. In-depth semi-structured interviews were conducted with 23 participants. Reflexive thematic analysis of the data revealed four major themes (1) entry and reentry visa restrictions for Iranians; (2) the emotional and social impact of the U.S. Travel Ban; (3) family separation and emotional distress; and (4) mental health impacts of immigration uncertainty. Findings highlight significant challenges faced by this population, including extended family separations, career uncertainties, and psychological distress. Participants reported heightened anxiety, insomnia, and feelings of isolation, aligning with research on migration-related mental health disparities. This research contributes to the limited literature on the experiences of Iranian immigrants in the U.S. and has implications for minoritized population health and policy reforms aimed at mitigating the adverse effects of immigration-related stress.

## Introduction

Immigrants make up 14.3% of the United States (U.S.) population [1–3]. While immigrant experiences are highly diverse, those from racialized backgrounds, precarious visa categories, or politically sanctioned countries have often faced structural disadvantages [4]. For these populations, health, particularly mental well-being, has been disproportionately influenced by the host country’s migration policies, which function as a critical social determinants of health [1–3]. In parallel, over one-third of immigrants aged 25 and older in the U.S. held a bachelor’s degree or higher in 2022, with 15% of them earning professional or doctoral degrees [4]– [5]. Among these, Iranians represent a distinct group with higher educational attainment compared to the overall immigrant population [6].

In 2019, the U.S. hosted approximately 385,000 Iranians, accounting for around 30% of all Iranians living abroad [7]. Other key destinations include Canada, followed by Germany, the United Kingdom, and Türkiye (Turkey) [6]. The growth in highly educated Iranian migration is linked to the complex political and socioeconomic factors. Since the 1979 Islamic Revolution, many Iranians have sought opportunities abroad to escape repression, corruption, and economic hardships [7].

However, Iranian immigrants living in the U.S. face unique challenges due to decades-long geopolitical tensions. Diplomatic tensions between the U.S. and Iran have remained persistent since 1979, when Iranian students took over the U.S. Embassy in Tehran and held 52 Americans hostage [5]. This event resulted in the severance of diplomatic relations and the permanent closure of the U.S. Embassy in Iran [6]. As a result, Iranian nationals must travel to third countries such as Turkey, Armenia, or the United Arab Emirates to apply for U.S. visas, creating a process that is both costly and burdensome [7]. These challenges were amplified in the years following the September 11 attacks, when the National Security Entry-Exit Registration System (NSEERS) was introduced. NSEERS targeted individuals from Muslim-majority countries, including Iran, for additional screening and monitoring [8]. The pattern of exclusion continued with the implementation of the 2017 travel ban, which sharply restricted entry for Iranian nationals and disrupted the educational and professional trajectories of many [9]. In 2018, the U.S.’ unilateral withdrawal from the Joint Comprehensive Plan of Action (JCPOA) and subsequent reimposition of sanctions further strained relations [10]. These policy shifts not only limited visa access but also disrupted financial transactions [10], compounding the challenges Iranian immigrants and students face in navigating life in the U.S.

In this climate, Iranian immigrants encounter a convergence of barriers resulting from both restrictive migration policies and strained bilateral relations. Immigration scholars have emphasized that such policies function as structural determinants of health, influencing mental well-being through mechanisms such as legal precarity, family separation, and prolonged uncertainty [1, 11]. Despite holding valid visas, many Iranian students and professionals experience substantial psychological distress due to administrative backlogs, limited visa mobility, and fear of traveling to Iran [12, 13]. For example, international students face extended background checks, delayed processing, and the emotional toll associated with single-entry visa restrictions [14].

These issues are especially pronounced in STEM-related sectors. Due to federal sanctions and export control regulations, Iranian professionals are often excluded from research involving sensitive technologies, discouraged from applying to national labs, or denied visa sponsorships altogether [15]. Institutional gatekeeping and fears surrounding knowledge-sharing between Iran and the U.S. further constrain the career mobility of Iranian scientists and engineers [15]. Moreover, immigration-related challenges lead to prolonged family separation, forcing many to choose between career advancement and family reunification [13].

It is often assumed that only undocumented or refugee populations are vulnerable to immigration-related stress. However, research increasingly showed that legally authorized immigrants, particularly highly skilled individuals, also experience adverse mental health effects [16]. Systematic reviews and meta-analyses estimate that migrants experience significantly higher rates of anxiety and depression than the general population, with pooled estimates around 20% for depression and 15% for anxiety [12, 17, 18]. These disparities are further compounded by underemployment, discrimination, and the loss of professional identity, which disproportionately affect educated migrants from politically sanctioned nations [13].

Among immigrant groups, Iranians have been identified as one of the most psychologically vulnerable populations. In the Netherlands, 43% of Iranian asylum seekers met diagnostic criteria for affective disorders, the highest among surveyed nationalities [19]. Similar patterns have emerged in Sweden, where Iranian immigrants report elevated levels of anxiety, depression, and psychotropic medication use compared to native-born populations [20, 21]. However, much of this research was confined to European contexts, narrowly focused on refugees or asylum seekers, and typically reliant on quantitative methodologies that overlook the lived experiences of individuals navigating complex immigration regimes.

This creates several critical gaps in the literature. First, the mental health consequences of migration policies for highly educated immigrants and international students remain underexplored, despite the distinct psychological burden posed by stalled visa processes, career disruptions, and limited mobility in professional fields such as STEM [15]. Second, although European studies have documented mental health challenges among Iranian immigrants, there is a lack of U.S.-based data. Policies specific to the U.S., including the “Muslim Ban” or “Travel Ban,” the absence of diplomatic representation in Iran, and prolonged administrative processing, contribute to a unique psychosocial environment that warrants targeted investigation. Third, much of the existing scholarship lacks qualitative insight into how highly educated Iranian immigrants interpret, cope with, and navigate these structural constraints in daily life [22].

### Theoretical Framework

This qualitative study employed a phenomenological approach to examine the lived experiences of highly educated Iranian immigrants and international students within the context of U.S. immigration policies, using the Social Determinants of Health (SDOH) framework [23] as an analytical lens. The SDOH model, developed by the World Health Organization (WHO), emphasizes that health outcomes are shaped not only by individual behaviors or genetics but by broader social, economic, and political conditions. For immigrants and international students, legal status, migration policies, and geopolitical contexts play a central role in structuring daily life, access to opportunities, and long-term well-being [24].

The SDOH framework is especially well-suited for this study because it enables a structural analysis of how policy environments shape mental health outcomes, even for immigrants who may appear socioeconomically advantaged. By framing migration policy as an upstream social determinant, this study situates legal precarity, visa restrictions, and family separation as systemic exposures that contribute to chronic stress, anxiety, and emotional distress. This framework enables a shift from individualized explanations of mental health to a structural understanding of how health inequities emerge, even among legally authorized and highly skilled immigrants. It also provides a validated lens through which to interpret participants’ narratives as reflections of structural vulnerability rather than personal inadequacy or cultural difference.

Applying the SDOH framework aligns this study with public health research that emphasizes the role of legal and political structures in producing health disparities. It reinforces the argument that immigration reform should be considered not only a legal or economic concern, but a matter of public health and equity.

### The Present Study

This qualitative exploratory study aims to address these gaps by examining how restrictive U.S. immigration policies function as social determinants of health among highly educated Iranians. This group represents a unique case within the immigration and international students’ landscape: while they often enter the U.S. through legal and merit-based pathways such as graduate education or skilled employment, their Iranian nationality places them under heightened scrutiny, delays, and exclusions linked to decades of geopolitical hostility. Although these individuals may have viable pathways to remain in the U.S. through employment or student visas, they frequently encounter structural and psychological challenges that threaten their long-term well-being. Through the lens of Social Determinants of Health framework, such immigration-related legal and institutional barriers are understood as upstream structural factors that shape downstream health outcomes, including chronic stress, anxiety, and emotional exhaustion. Highly educated Iranian immigrants and international students may be especially vulnerable because, despite their academic credentials and legal status, they are often excluded from advocacy efforts or institutional support, leaving them isolated and overlooked. Their perceived privilege may mask the psychological harm caused by exclusion from sensitive research settings, single-entry visa restrictions, and limited transnational mobility—all of which function as social determinants with direct implications for mental and emotional health. By centering lived experience, the study offers a deeper understanding of how immigration policies become embodied as health inequities.

## Methods

The study was reviewed and approved by the Institutional Review Board of Portland State ] University. This paper draws on an interpretative phenomenological study that explores the anonylived experiences of highly educated Iranians, focusing on how restrictive U.S. immigration policies function as social determinants of health. Guided by the principles of Interpretative Phenomenological Analysis (IPA), the research team aimed to understand how participants make sense of their encounters with U.S. immigration policies and to interpret patterns of meaning both within individual narratives and across cases.

### Participants

A total of 23 highly educated Iranian immigrants and international students were recruited for the study through convenience sampling, following the advertisement of a study flyer in two online immigration forums. The flyer included information such as the study title, IRB number and affiliated institution, purpose of the research, what participation involves, eligibility criteria, and information about compensation. It also featured a QR code and a link to a short demographic survey, which allowed interested individuals to provide their email addresses to be contacted for an interview. Additionally, the flyer listed the principal investigator’s email address, and some participants reached out directly to express interest in participating.

Inclusion criteria required participants to be adult, first-generation Iranian immigrants or international students, defined as individuals born in Iran who migrated to the U.S. as adults and held either a valid visa or permanent residency at the time of the interview. Participants were also required to be highly educated and both able and willing to participate in an audio-recorded Zoom interview conducted in English. For this study, “highly educated” referred to individuals who were either pursuing or had obtained a graduate degree from a U.S.-based university at the time of the interview. Exclusion criteria included individuals who were unable to attend a Zoom interview or declined to provide consent for audio recording. In this study, highly educated Iranian immigrants and international students were analyzed as a single group. Although international students typically hold temporary non-immigrant visas, they were included under the broader category of immigrants due to their common expressed intent to establish long-term legal residence, active navigation of the U.S. immigration system, and common lived experiences shaped by restrictive immigration policies.

### Data Collection

The study was reviewed and approved by the Institutional Review Board at [Portland State University. All participants provided written electronic informed consent in English prior to participation. Due to COVID-19-related restrictions, interviews were conducted via Zoom between April 2021 and March 2022. The interviews were conducted in English using a semi-structured protocol designed to facilitate open-ended exploration of participants’ migration experiences, academic and occupational trajectories, and the impacts of U.S. immigration policy on their livenonys. No identifiable information was collected during the interviews. A trained research assistant, supervised by the second author, conducted all interviews. The interviewer did not share the participants’ cultural or national background, an intentional choice to further support the confidentiality of the participants. We used member checking during the interviews to confirm our understanding of participants’ responses, clarify meanings in real time, and adjust interview prompts as needed.

Interviews were audio-recorded and transcribed using Zoom’s transcription feature. As an added measure of confidentiality, participants were asked to keep their cameras off. Only audio recordings were downloaded, and video files, automatically generated by Zoom, were deleted immediately after each interview. Audio files were stored along with the Zoom-generated transcripts on the password-protected laptops of the interviewer and the second author. Transcripts were then reviewed and edited by them to ensure accuracy and to remove any identifying information. Audio recordings were promptly deleted once the transcripts were finalized. Interview durations ranged from 26 to 75 min.

### Analysis

Qualitative data were analyzed inductively and deductively using reflexive thematic analysis [25]. The research team engaged in an iterative process of reading and re-reading the transcripts. During this phase, five transcripts were open-coded by the first and third authors independently. The open codes generated were subsequently reviewed by the second author, who consolidated similar codes to develop a comprehensive codebook. This codebook was then used to code the remaining interviews. Once all transcripts were coded, the second author reviewed the coded data and generated a series of proposed themes. These themes were discussed and refined in collaboration with the research team until a final set of themes was agreed upon. To support each theme, relevant quotes were extracted from the transcripts, with two to three quotes selected for inclusion in the paper to illustrate and substantiate the findings. Throughout the coding and theme development process, special attention was paid to negative case analysis to enhance the rigor of the study.

## Results

Participant demographic characteristics, including age, gender, education, marital status, income, visa type, and length of stay, are summarized in Table 1. The sample consisted predominantly of males (65%) and individuals holding doctoral degrees (73%). Most participants were married (56%) and had resided in the U.S. for 5–6 years (61%). The majority reported an annual income between $25,000 and $100,000. In terms of immigration status, 60% held student, OPT, or dependent-to-student visas, while a smaller proportion were permanent residents (22%) or on work visas (13%).

**Table 1:** Demographic characteristics

Characteristic	Category	*n*	%
Age	28–36 years (M = 32.7)	–	–
Gender	Male	15	65
	Female	8	35
Education level	Doctoral degree	17	73
	Master’s degree	6	27
Marital status	Married	13	56
	Not married	10	44
Annual income†	<$25,000	3	13
	$25,000–$50,000	7	30
	$50,000–$100,000	9	39
	$100,000–$200,000	4	17
	>$200,000	2	1
Current visa status	Student/OPT/Dependent to student visa	14	60
	Permanent resident	5	22
	Applying for permanent residency	1	4
	Work visa (H-1B or H-4)	3	13
Length of stay in U.S.	2–4 years	9	39
	5–6 years	14	61

OPT = Optional Practical Training; H-1B = temporary work visa; H-4 = dependent of H-1B visa holder

†Income data are based on the subset of participants who reported income; percentages may not total 100 due to rounding.

Four major themes emerged from qualitative analyses of the data: (1) Entry and reentry visa restrictions for Iranians (2) The emotional and social impact of the U.S. Travel Ban (3) Family separation and emotional distress and (4) Mental health impacts of immigration uncertainty.

### Entry and Reentry Visa Restrictions for Iranians

Across the interviews, a recurring theme was the prolonged and complex procedures for securing an entry visa due to restrictive migration policies. Interviewees expressed a sense of inequity and discrimination in the application process for an entry visa to the U.S., with many feeling that their nationality subjected them to additional scrutiny and barriers that others did not face. For instance, an Iranian doctoral student (interviewee #13) shared, “Especially being from Iran, I have specifically felt some stuff in terms of like background check, just, like, getting the visa to come in here, like, they were super long.” Interviewee #11, also a doctoral student, explained the added challenges faced by Iranian applicants for an entry to the U.S., who must travel to neighboring countries to secure a visa due to the absence of a U.S. embassy in Iran. She said “We don’t have a United States embassy in our country, so we should travel to another country to have [a] visa interview.” Another student (interviewee #15) provided further context of difficulties associated with in-person interviews in the absence of a U.S. embassy in Iran in getting student visas to come to the U.S.:

*After the visa appointment I had in Turkey*,* I went back to Iran and waited a month until they announced whether it [was] approved or not… I actually missed the flight I booked to the U.S.*,* so I had to cancel that flight and book a different flight directly from Turkey to [the] U.S. So I went to Turkey*,* picked up my visa*,* which took like a couple of days*,* …*,* yeah*,* I*,* I*,* I had to go like*,* to Turkey for a week before moving com- completely to the U.S.*

Interviewees described wait times ranging from a month to over a year while waiting for their entry visas to be approved and their background checks and clearance processes to be completed. For instance, one interviewee, interviewee #17, shared that he waited 14 months after his interview to receive his student visa. He told us:

*When I applied for the non-immigrant visa for my student case*,* it also took*,* oh*,* 14 months. I’m not sure if [it was] due to the travel ban or not*,* but it may be*,* yeah…. I got admission [in] 2018*,* but- and applied for the visa*,* but it [took] 14 months*,* so I postponed my admission for two semester[s]*,*… it was hard.*

The single-entry visa restriction for Iranian students also emerges as a significant concern, preventing them from visiting their families or traveling without the risk of being unable to return to the U.S. Many interviewees shared that they have been unable to see their parents or loved ones for extended periods—sometimes for several years—due to the restrictions imposed by their single entry visas. This prolonged separation has caused considerable emotional distress and negatively impacted their mental health. For instance, a married doctoral student on a student visa (interviewee #13) expressed, “It’s really hard since I haven’t seen my parents in five or six years…. I was on a single-entry visa.” Another student (interviewee #15) explained, “I’m from Iran, so my F-1 [student] visa was single entry, and I haven’t been able to go back for seven years.” Similarly, an interviewee on an Optional Practical Training (OPT, interviewee #21), which is an extension to a student visa allowing temporary employment, reflected on the cumulative emotional toll of being on a single-entry visa, saying:

*It’s been six years… I haven’t seen my family. When you come to the United States for a PhD*,* you’re often in your 30 s or even 40s*,* and that means your parents are older. You’re constantly worried about them… I was told my mom had heart surgery. I have friends who have lost loved ones and just didn’t know what to do.*

A few interviewees had a different experience as they received multiple-entry visas for the first two years of their stay in the U.S., but even they experienced family separation by constrained choices after the first two years and expiration of multiple entry visa. One student (interviewee #17) shared that although he had a multiple-entry visa, he was unable to take advantage of it due to the COVID-19 pandemic: “I entered the U.S. in 2019, and my [multiple-entry] visa expired after two years. And due to the COVID pandemic, I couldn’t use my visa, although it was multiple entries.”

Interviewees explained that, although it was technically possible to leave the U.S. on a single-entry visa and reapply for a new one to return, they were hesitant to do so. The fear of being denied a new visa or facing prolonged processing times created significant uncertainty, potentially jeopardizing their education or work. Consequently, many opted to remain in the U.S., leading to extended periods of separation from their families. For instance, one Iranian student (interviewee #3) pursuing a master’s degree on a single-entry visa described his experience as:

*We have a single-entry visa. It means that when we come to [the] U.S.*,* if we want to leave*,* we need … to go through the whole process*,* and there’s a chance that we don’t get a Visa the second or third time. And exactly I saw the same thing happen to two Iranian[s] that came to the embassy same day as I was there … they were returning student[s]*,* they came to Iran for*,* like*,* wedding ceremonies*,* or some illness in their family*,* or something like that…. they didn’t get the returning visa.*

Despite the challenges associated with restrictive immigration policies related to entry visa issuance and reentry, there were recurring statements of hope and resilience among the interviewees. Many interviewees shared their determination to work hard toward their goals, holding onto the hope that they will eventually reunite with their families. However, some participants also questioned whether the sacrifices, including years of separation from loved ones, are truly worth it. Interviewee #20, who had not seen his parents for over eight years, reflected on this uncertainty: “So these just cumulatively adding together and making you say that, ‘Did I select the right path? I don’t know.’… My wife … she’s been also eight years haven’t seen her family.”

#### The Emotional and Social Impact of the U.S. Travel Ban

Several participants discussed the difficulties they faced as a result of the travel ban, which some described as creating feelings of isolation and uncertainty. One female participant with work visa approval (interviewee #23) shared her experience during the travel ban, saying: “I was feeling that I’m present in a big and sometimes beautiful, attractive prison. I’m working and I’m paid as a prisoner, but I’m not allowed to go to even have a visitor. And I’m not allowed to even just take a break for a day.” Another female doctoral student with a pending permanent residency application (interviewee #22) described the uncertainty caused by the travel ban and the emotional difficulty of being separated from her family.

*My family couldn’t come to the U.S. because of [the] travel ban- no one can come to the U.S.*,* so I was stuck here for four years*,* not being able to see any- and I was here alone by myself*,* so I didn’t have anyone other than a couple of friends that I made*,* I made a friendship with them here in the U.S. … I didn’t know them before. So*,* I was all by myself… very tough … full of uncertainty.*

Other participants shared similar experiences of stress and uncertainty related to the travel ban. One married male doctoral student in the process of obtaining permanent residency (interviewee #9) described the stress and uncertainty caused by the travel ban, noting the hope that he might someday be able to invite his family to visit.

*I mean it has been a very stressful four years*,* last four years starting from the travel ban*,* or the Muslim ban which- Yeah*,* we couldn’t- we knew that when we applied*,* when we came here*,* we knew that we are not going to be able to exit the country [because of the single entry visa] but we- at least we had this in the back of our mind*,* okay*,* so some days*,* like*,* we might be able to send invitation for our parents to come and visit us*,* and that would be a possibility that*,* through that*,* we can visit our parents*,* but starting there*,* like- yeah*,* you know*,* to- we realized*,* okay*,* we can’t even- we are not going to be able to see our families probably for undisclosed amount of time until this ban is going to be lifted. So yeah*,* I mean*,* every day*,* we have to just hope that nothing worse happens….*

Some interviewees described facing an emotional toll due to the combination of the travel ban and the pandemic. One male doctoral student (interviewee #14) shared that he lost multiple family members during COVID-19 and was unable to visit them due to the travel ban. He expressed uncertainty about when, or if, he would be able to see his family again, despite hoping that the travel ban might be lifted.

*It’s been almost five years [since] I haven’t visited my family… during the past administration*,* they put this ban [on a] few countries*,* including Iran*,* so I couldn’t even invite my family. So- and I don’t even know when I- when I would be able to visit my family. I- during the past year*,* during COVID*,* I lost three people in my- in my family*,* not*,* like*,* my parents*,* just*,* like*,* my uncle and two- two more people. I lost my… grandfather*,* and … I couldn’t go back and be at their funeral and say goodbye… I know that the administration changed and they’re talking differently*,* but still*,* there is no sign of any change … in [the] Iran and U.S. relationship.*

Some participants mentioned that they stopped sharing their personal hardships and experiences with family members back home to avoid causing them additional stress and guilt during the travel ban. One female participant with permanent residency (interviewee #18) explained, “When I got cancer, and I was in hospital…, I didn’t tell anyone because, like, they cannot come here, why should I make them stress? I told her [my mom] like, …, nine months later… I didn’t tell her what, I said it was something that was not bad… she still doesn’t know, and then she started crying …but she, she was like, I’m glad that you are okay.” Another female participant with work visa approval (interviewee #23) shared a similar experience, describing how the travel ban affected her family in Iran:

*I had three miscarriages… that was really tough for me… I could not share it with my mother because I was just feeling that… I just didn’t want to add extra stress on her … So there were lots of issues with [the] travel ban … I hope- I’m very happy that they have no new travel ban…my parent*,* I mean our parents- mine and my husband’s were really sad… Particularly during the COVID because … my father was impacted*,* my husband’s mother was impacted… that was very bad.*

A few participants discussed missing important family moments due to the travel ban. One female participant with permanent residency (interviewee #18) shared that she was unable to be with her family for significant life events: “I couldn’t, like, ten, eight years, I didn’t see my family. I lost, I missed all the important times, you know…I missed my sister’s wedding… when she had a baby, and then all those times I missed it, and I cannot take it back.” Another married male participant with a doctoral degree in a medical field with permanent residency (interviewee #2) described the social impact of the travel ban on his family: “My older son is now almost four years old, and he hasn’t met my parents because of the travel ban. And, even though it’s lifted now, it’s the Covid situation that doesn’t allow any, you know, temporary visitors. So, that was a huge impact socially on me … I haven’t seen my parents for, like… more than six years.”

The uncertainty of the travel ban led some participants to face difficult decisions about whether to stay in the U.S. or risk leaving and being unable to return. One male participant (interviewee #21) shared his concerns about traveling back to Iran, questioning whether he would be able to return due to the ban.:

*Okay*,* what if I go to Iran… I cannot come back because of the ban. While a lot of students were able to come in maybe a couple of months after the ban was placed*,* it was mostly psychological. It did have some real implications for the flow of students to the United States. Students didn’t want to risk going back to Iran because they feared they wouldn’t be able to come back.*

This sentiment was echoed by a married male participant (interviewee #3), a master’s degree holder who applied for OPT. He explained:

*The community of students here*,* they*,* we all understand what’s happening to us*,* especially if they’re Iranian because*,* like*,* we are kind of different. We cannot leave*,* leave here to visit our country because you don’t*,* like*,* … you’re*,* like*,* spending the same three years of your life for PhD and then you leave for something*,* and you cannot come back and finish your PhD*,* for example. Yeah*,* so usually people [don’t] leave*,* especially after Trump came.*

Some participants mentioned knowing friends or hearing stories of people who left the U.S. on a visa intending to return but were unable to, particularly during the travel ban. Interviewee #21 told us: “I cannot come back because of the ban…I know such cases that people left and there were stuck in Iran only because of the ban.”

#### Family Separation and Emotional Distress

Participants described the prolonged separation from loved ones back home as a profound psychological burden, with several graduate students noting that this isolation adversely affected their academic performance and overall well-being. Being unable to visit family for extended periods, sometimes up to a decade, caused immense distress, sadness, and strained interpersonal connections. As interviewee #7 expressed, “it reduces your patience and…whenever you’ve got a challenge in your education or you have a challenge with…anything here, that’s…not being able to be…with your family in the background, would I guess double, you know, the pain.” Similarly, a male doctoral interviewee (interviewee #17) indicated, “I almost were with my family for 20- the previous 25 years, but right now, during these 3 years, I haven’t seen them. I’m not able to, you know? And it almost impacts my study or my [focus].”

#### Mental Health Impacts of Immigration Uncertainty

Participants recounted periods of severe depression, insomnia, and panic attacks as they grappled with the vague and unpredictable process as immigrants and international students. For instance, a doctoral student with a permanent residency application under review (interviewee #4) said, “I’m taking five medicines, every day…I have some problem[s]; I feel trembling when I sleep… they told me that that’s probably because of the interaction with my vast number of medicines for anxiety and depression.” Interviewee #22, a female pharmacist pursuing her NIW shared: “when I think about doing something here I’m in limbo- it’s full of uncertainty, I’m just- I’m just like living my daily life on a daily basis because I’m not sure about the future at all and it’s very frustrating. I cannot plan, like, having a long-term plan for myself.” Interviewee #19 described the experience as being “under a lot of pressure” that “double[d]” the normal challenges they faced. The ever-present fear of potential deportation or denial of status compounded this distress, with participants reporting high relevant stress levels. More than half rated their stress above 7 on a scale of 1 to 10, with 10 being the highest. For example, a married doctoral student (interviewee #9) explained about the urgency of finding a job to maintain legal status after graduation and told us:

*If you cannot find [a] job after three months*,* once you are on OPT then- then that’s it*,* you’re out of status*,* so. It’s still a very stressful situation*,* so it- because you should- you should look for a job after you’re finished [with your education]*,* you are forced to make a decision*,* and like find the first- usually you should go for the first thing you find*,* you don’t have time to collect yourself after… I’m finished*,* I have*,* like*,* three months to find a job … if I don’t find jobs within three months then I’m out of status. So this is one thing that- yeah… PhD life is a little bit… stressful*,* just knowing these time limitations down the road.*

Importantly, the emotional strain did not solely impact the immigrant participants but also extended to their spouses and families. Participants described how their own anxiety and depression strained their relationships and placed an emotional burden on their loved ones. As a married doctoral student (interviewee #7) shared, “It impacts our relationship as well. And we actually…whenever we had…an [argument] or so, we…think that okay, this is because we are…tolerating so [many] challenges, and that’s why we are not patient anymore.” The uncertainty and restrictions also caused immense worry for the family members of participants back home, specifically as they were separated by constrained choices. For example, interviewee #18 shared her experience while she was waiting in uncertainty for her work visa (H1), “She [my mom] was in constant stress, I can say too, because I wasn’t as stressed.”

## Discussion

The demographic profile of the study participants aligns with existing research on highly educated Iranian immigrants and international students in the U.S. The sample was predominantly composed of young, highly educated individuals, with the majority holding doctoral degrees and the remainder having master’s degrees [26]. The gender distribution, with slightly more male participants, is also consistent with the broader population of Iranian immigrants and international students in the U.S [26].

The first major theme that emerged from the interviews highlighted the struggles faced by highly educated Iranians in navigating the complex and restrictive entry visa application process and the persistent fear of being denied reentry into the U.S. Participants described lengthy delays, additional security checks, and the challenges of obtaining visas in the absence of a U.S. embassy in Iran. These hurdles were not experienced as mere administrative inconveniences to enter the U.S.; rather, participants viewed them as targeted obstacles disproportionately affecting specific nationalities, particularly Iranians, contributing to a sense of exclusion. These findings resonated with existing research that has documented the significant barriers and uncertainty experienced by Iranian students in the U.S. immigration system [14, 15]. The prolonged wait times and fear of being denied entry upon return can have profound emotional and financial consequences, as evidenced by participants’ experiences of missed academic and professional opportunities.

Participants’ experiences reinforce prior work on legal liminality and visa-based immobility among nationals from sanctioned countries [1, 14, 27, 28]. Yet, this study sharpens that lens by showing how even legally authorized, highly educated individuals face prolonged separation and emotional harm. In this way, we argue that restrictive visa regimes for Iranian nationals function as a form of state-sanctioned immobility [27], where legality coexists with constrained mobility and chronic uncertainty; key mechanisms through which structurally embedded policies generate health inequities, not through exclusion alone, but by normalizing disruption and disconnection. The second theme underscored the profound impact of the U.S. Travel Ban on Iranian immigrants and international students. Participants described how the ban enforced prolonged family separation, preventing visits from parents and spouses for years. As further explored in the third theme, this sustained separation contributed to depression, anxiety, and strained interpersonal relationships. These findings aligned with previous research highlighting the detrimental impact of family separation on the mental health outcome among immigrant populations [12]. Participants’ concealment of illness, miscarriage, or grief reflects what Gross [29] terms “response modulation”, a late-stage emotion regulation strategy often triggered under constrained conditions. The chronic inability to socially share emotions [30] deprives individuals of a key stress-buffering mechanism and is linked to increased anxiety, depressive symptoms, and social disengagement [31, 32]. Given that family cohesion is known to buffer against stress [33], our findings further support calls for immigration policies that protect family unity. Efforts such as simplifying re-entry procedures, offering multi-entry visas, and easing visit permissions for immediate family members could help reduce the long-term psychological and physical harms associated with sustained or anticipated separation [2, 34, 35]. These findings are particularly relevant to the current immigration landscape, where the 2025 version of the U.S. Travel Ban continues to disproportionately impact Iranian immigrants and international students and prolong separation from close family members, compounding the psychological toll.

The final theme highlights the emotional and psychological burden created by immigration uncertainty, emphasizing that immigration policies function as structural determinants of mental health for Iranian immigrants and international students. Participants frequently described feeling as though their lives were “on hold,” unable to make long-term plans due to the unpredictability of their legal status. Fear, particularly fear rooted in immigration policies, emerged not as a fleeting emotion but as a persistent, organizing force that shaped their behaviors, decisions, relationships, and overall well-being. The psychological burden of uncertainty and prolonged wait can manifest in mental health issues such as anxiety, insomnia, and depression. Research in stress and anxiety studies highlights that prolonged uncertainty triggers the brain’s heat-detection systems, putting individuals in a continuous state of alert [36]. Building on foundational models of stress and control [37, 38], our findings showed that prolonged immigration uncertainty systematically impairs executive functioning, goal pursuit, and emotion regulation; psychological processes essential not only to individual well-being but to the very productive potential that host countries seek to attract through skilled immigration.

These findings emphasized the need to consider immigration-related stress as a critical social determinant of health [13]. Our previous research on highly educated immigrants further emphasized that restrictive immigration policies are closely linked to significant health disparities and inequalities [2]. These findings challenge the perception of highly educated immigrants as a uniformly resilient or privileged group by showing how even immigrants with advanced education face profound emotional hardship under restrictive immigration policies.

### Strengths and Limitations

This study has several limitations that should be considered when interpreting the findings. The sample had limited gender diversity, with 65% of participants being male, which may limit transferability to more gender-balanced populations. Furthermore, the study was conducted during the COVID-19 pandemic, which may have exacerbated certain challenges and added additional layers of complexity to the participants’ experiences. Additionally, conducting interviews via Zoom may have introduced power imbalances or limited rapport between interviewers and participants, which is a known challenge in online qualitative interviewing. Despite these limitations, the in-depth, phenomenological approach provided valuable insights into the lived experiences of this understudied population, highlighting the significant impact of restrictive migration policies on their well-being.

### Conclusions and Recommendations

This study offers a critical exploration of the unique challenges faced by highly educated Iranian immigrants and international students in the U.S., highlighting how restrictive migration policies serve as social determinants of health for this population. While the U.S. continues to be a primary destination for Iranians, particularly those with advanced degrees, their experiences are shaped by systemic barriers that create significant emotional, psychological, and professional hurdles.

Based on participants’ narratives, even modest improvements in immigration policies related to entry visas, such as increased transparency and clearer communication during the application process, could significantly reduce the stress and anxiety associated with obtaining a U.S. visa. Granting students multiple-entry visas, rather than single-entry ones, could also enhance their well-being and academic performance by alleviating the persistent fear of being unable to return if they leave the country. In cases where multiple-entry visas were issued, they were often limited to just two years, despite the longer duration of doctoral programs. Aligning the length of multiple visa entry validity with the expected duration of academic programs could better support the growth and mental health of international students.

Our findings suggest that the U.S. Travel Ban contributed to prolonged family separations, which in turn led to heightened anxiety, depression, and emotional distress among participants. The use of family separation as a tool of oppression or deterrence against specific immigrant groups is not new in the U.S. context [39]. However, policies resulting in family separation should be critically re-evaluated due to their psychological harm. Immigration frameworks must account for the fundamental importance of maintaining family connections. Mechanisms should be in place to allow immediate family members to be able to visit and maintain relationships.

Our findings also highlighted the mental health burden of immigration uncertainty. Immigration agencies and official sources should consider providing timely updates and predictable timelines for application processing to reduce prolonged uncertainty and the associated psychological distress among immigrants. Organizations working with immigrants and international students, including universities, could partner with legal aid services to help immigrants and international students better understand and navigate their immigration options, reducing confusion and fear about their status. Future research should also consider how support systems embedded in universities, communities, or legal institutions might be able to support immigrants and international students to alleviate the psychological toll of restrictive immigration policies. Investigating whether immigrants’ and international students’ experiences differ by personal or demographic factors, such as marital status, gender, or visa category, may also help refine targeted responses and inform advocacy for more equitable policy reforms.

## Data Availability

No datasets were generated or analysed during the current study.
